# Ganglion of the Sternoclavicular Joint: A Rare Location of an Uncommon Pediatric Mass

**DOI:** 10.1155/2020/2035756

**Published:** 2020-03-26

**Authors:** Jacob Musiol, Jacob Hansen, Jonathan Wood

**Affiliations:** Department of Radiology, MCHK-DR, Tripler Army Medical Center, 1 Jarrett White Road, Honolulu, HI, USA

## Abstract

This is a case of a two-year-old boy with a ganglion arising from the sternoclavicular joint. Ganglia are rare in the pediatric population, with less than 2% occurring under the age of 2 years old. Additionally, sternoclavicular joint ganglia are also exceedingly rare. The case illustrates the importance of keeping ganglion within the differential diagnosis for palpable subcutaneous masses, even in young children, especially when they are seen to connect to the joint.

## 1. Case Presentation

A two-year-old boy was referred to Radiology for sonographic examination of a palpable mass overlying the left sternoclavicular joint. The mass was found one month prior to presentation and had been slowly increasing in size. According to the mother, the patient did not complain of any pain or pruritis, and the only symptoms appreciated were intermittent low fevers which began after the mass was noticed. Lack of symptomatology thereby led to the delay in presentation to a healthcare provider. After one month without regression, the patient's parents decided to seek medical care.

On physical exam, the mass was noted at the superior aspect of the left sternoclavicular joint. Palpation elicited no pain and demonstrated a mobile, firm, mildly compressible subcentimeter bump. A chest X-ray was performed which demonstrated no lytic or blastic lesions of the sternum, clavicle, or scapula.

There were no radiopaque soft tissue densities or ectopic calcifications noted, and the joints themselves were normal in appearance. No masses were visualized in the superior lobes of the lungs. CBC, ESR, LDH, and CRP showed no abnormalities.

**Figure 1:**
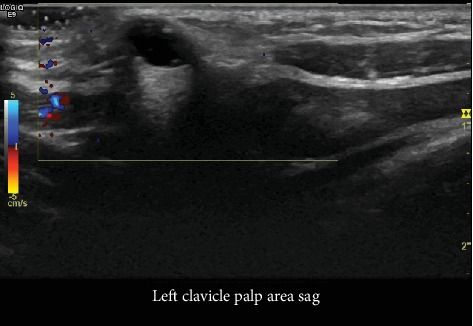
Single sonographic image with color Doppler of the left sternoclavicular joint demonstrates a circumscribed, avascular, cystic mass with a narrow neck that dives into the sternoclavicular joint. This connection with the joint is typical for ganglia.

**Figure 2:**
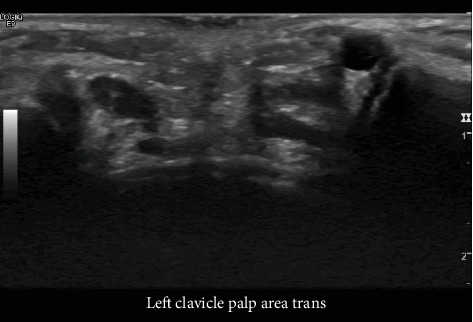
Single sonographic image of the right and left sternoclavicular joints shows a circumscribed, round, cystic mass with increased through transmission located just anterior to the left sternoclavicular joint.

Ultrasound imaging was performed which demonstrated a 6 × 4 × 4 mm irregular anechoic, thin-walled structure, without internal color Doppler signal, and with increased through transmission consistent with a cyst which demonstrated a narrow neck diving deep into the left sternoclavicular joint (Figure 1). There were no internal septations, debris, or solid components (Figure 2). These findings were consistent with the diagnosis of a ganglion of the sternoclavicular joint. After discussing the imaging and benign nature of the disease, the patient's mother elected for orthopedic referral due to cosmetic concern. The orthopedic surgeon deferred removal of the ganglion at this time given the lack of pain or alterations in shoulder motion and elected for simple observation in the absence of changes in clinical presentation.

## 2. Discussion

Ganglia, also referred to as ganglion cysts, are fluid-filled soft tissue swellings that occur most commonly over the dorsal surface of the wrist. These ganglia can occur at any point in life although their incidence is the highest between the second and fourth decades [1]. Cases of pediatric ganglia are notably rare with one study citing that 10% of ganglia occur in patients under the age of 20 and less than 2% occur in children under the age of 10 [2]. In the general population, 70% of ganglia are reported to be located on the dorsal wrist with another 20% on the palmar aspect of the hand [1]. Sternoclavicular ganglia fall within the remaining 10% of miscellaneous anatomic sources. The combination of a two-year-old patient with an uncommon anatomic location such as the sternoclavicular joint makes for an exceedingly rare case.

Ganglia are most often characterized by a communicating pedicle between the lumen of the cyst and the synovium of their source joint [3]. It is theorized that the fluid within the cyst demonstrates unidirectional flow due to a one-way valve mechanism. Studies have demonstrated this through injection of contrast dye into the cyst with no observed retrograde flow into the affected joint.

Conversely, injection of contrast into the joint space led to anterograde flow of contrast medium into the cystic space [4]. The fluid within a ganglion is distinct from synovial fluid with high concentrations of hyaluronic acid, whereas the cyst itself is not contained by a synovial membrane [3]. This distinguishes a ganglion from a synovial cyst, another common type of soft tissue mass.

Ganglia can be preliminarily diagnosed with physical exam through transillumination of the mass. A ganglion will transilluminate due to its fluid composition in comparison to a solid mass which will not. Ultrasound imaging is the fastest and simplest imaging modality for diagnosis with MRI functioning as a confirmatory method if ultrasound is equivocal. Typical ultrasound findings demonstrate anechoic, cystic architecture with well-defined margins and posterior enhancement due to the fluid-filled nature of the cyst [4, 6]. In a study by Teefey et al., it was shown that most ganglia are complex in nature with internal septations being common, especially in cysts of the palmar and dorsal wrist. Simple cystic architecture was more often found in cysts of the flexor tendon sheath. It is of note that the architecture of the cyst in this case report is also simple which further accentuates the rarity of the patient's condition.

Additional findings noted by Teefey et al. include the presence of internal reflectors and vascular flow feeding the cystic tissue [5]. The presence of solid components leads away from but does not exclude the diagnosis of a ganglion as solid masses can occur and often mimic benign soft tissue neoplasms [5].

The use of MRI is largely reserved for cases of equivocal sonogram findings accompanied by a high degree of clinical suspicion for a ganglion. Findings corroborate those seen on ultrasound with cysts appearing as unilocular or multilocular fluid collections adjacent to the affected joint. On T1 imaging, the cysts will normally demonstrate low signal although the presence of various proteinaceous effusions or bleeding into the cyst may intensify the signal. On T2 imaging, the cysts predictably contain a high-intensity signal due to their high fluid content [6].

In conclusion, ganglia are a relatively common type of soft tissue mass in the adult population, most commonly occurring about the joints of the wrist. Although the imaging characteristics of the ganglion described in this case report are typical, it is particularly rare given the atypical joint involvement and the young age of the patient.
